# Impact of structural-level environmental interventions on physical activity: a systematic review

**DOI:** 10.1007/s00420-023-01973-w

**Published:** 2023-04-26

**Authors:** Edgar D. Hernández, Elisa A. Cobo, Lawrence P. Cahalin, Pamela Seron

**Affiliations:** 1 Facultad de Medicina, Human Movement Department, Universidad Nacional de Colombia, cra 45 30–00, Bogotá, Colombia; 2 Facultad de Ciencias de la Salud, Universidad de Boyacá, Boyacá, Tunja Colombia; 3 Department of Physical Therapy, University of Miami, Miami, Florida USA; 4 Facultad de Medicina, Universidad de La Frontera, Claro Solar #112, Temuco, Chile

**Keywords:** Environment, Built environment, Physical activity, Active transport

## Abstract

**Objective:**

To determine the effectiveness of structural-level environmental interventions on the changes in PA levels in the populations examined.

**Methods:**

Natural experiments that involve environmental intervention with structural modification were included. The primary outcome PA levels with consideration of both objective and subjective measurements. An electronic search was carried out in Medline/Pubmed, SCIENCE DIRECT, WEB OF SCIENCE, and CINAHL up to January 2022. Two reviewers screened titles and abstracts, selected studies, extracted relevant data, and examined study quality. A qualitative synthesis was performed.

**Results:**

Twenty-six articles were included. The structural-level environmental interventions included 4 fundamental areas: schools, work environments, streets or cities, and neighborhoods or parks. Of the 26 studies, 21 examined outdoor environments like parks, cities, pedestrian walkways, or steps, while 5 examined indoor or closed environments like schools and workplaces revealing that structural-level environmental interventions improve PA levels with the greatest effect in parks and active transportation. A risk of bias is inherent in natural experiments which is a limitation of this study. In schools and work areas, there is evidence of both decrease in sedentary time and an increase in PA related to environmental modifications.

**Conclusion:**

Structural-level environmental modifications in parks and active transportation demonstrated greater effects in promoting PA. Environmental modifications can impact physical activity in the population. Given that the economic and cultural setting is a key variable when considering the effectiveness of structural interventions, and since only 1 of the 26 reviewed articles included such data, more studies examining economic factors are needed especially in low- and middle-income countries like in South America.

**Trial registration:**

PROSPERO CRD42021229718.

**Supplementary Information:**

The online version contains supplementary material available at 10.1007/s00420-023-01973-w.

## Background

Physical activity (PA) is defined as any movement produced by the skeletal muscles that require energy consumption or modification of the metabolic rate (Vidarte et al. 2011). It is used worldwide as a strategy to promote health, reduce the burden of disease and improve the quality of life in populations (World Health Organization 2018) throughout the life cycle (Bidzan-Bluma and Lipowska 2018; Lees and Hopkins 2013; Prakash et al. 2015), and aid the prevention and treatment of chronic diseases such as hypertension, diabetes, heart disease, osteoporosis, chronic obstructive pulmonary disease (COPD) and Parkinson’s disease (Billinger et al. 2014; Mantoani et al. 2017).

Interventions or strategies that promote PA can be classified at either the individual or community level. Where such strategies are part of public policies, the evidence suggests they have a significant positive effect on PA levels (Pratt et al. 2014; Shi et al. 2017). Some interventions that are environmental at the physical level, such as modifications to parks, bicycle paths, cycle lanes, areas and spaces at work or in schools, industrial areas or transportation systems, are understood as strategies to promote healthy lifestyle habits in the individual but not necessarily the community (Chow et al. 2009; Stankov et al. 2020). The theoretical concept of purposely designed and built environments to facilitate PA makes it clear that the promotion of PA depends not only on the individual but also on the surrounding environment (“Overview | Physical activity and the environment | Guidance | NICE,” n.d.). Thus, having schools with activity spaces for the children implies greater PA and having fewer obesogenic environments impacts adults and older adults´ sedentarism (Seefeldt et al. 2002; Corsi et al. 2013; Sisson et al. 2019). In this vein, the development of policies and programs in the nations that stimulate PA have been proposed as strategies at the population level (Overview | Physical activity and the environment | Guidance | NICE n.d.).

Because of the above, global environmental structural modifications have been implemented as a public policy measure in several countries with a key method to measure their impact being through natural experiments (Craig et al. 2012). The natural experiment as a research study has two characteristics including the examination of the natural variation of an exposure an outcome (Craig et al. 2017) and the intervention takes place in the natural environment without the intervention of the researcher (Leatherdale 2018).

The evidence from natural experiments developed in various contexts (Risica et al. 2019; Mancipe Navarrete et al. 2015; Hernández-Álvarezet al. 2015; Sandu et al. 2018) examining the effect of structural-level environmental interventions on promoting PA (Becerra et al. 2013; Sugiyama et al. 2014; Alexandre 2013; Alexandre 2013) is widespread making it necessary to aggregate and review these data. This systematic review aims to determine the effectiveness of environmental interventions with structural or physical modifications on the changes in the population’s PA level. Our hypothesis is that structural or physical modifications at the environmental level lead to an increase in the PA level in the population.

## Methods

This study is a systematic review conducted according to Cochrane Collaboration (Deeks et al. 2011) and PRISMA (Moher et al. 2009) guidelines. The protocol is registered in the PROSPERO international database of systematic reviews under the number CRD42021229718.

### Selection criteria

#### Type of studies

Natural experiments that involve structural- or physical-level environmental intervention programs to promote PA. “Understanding a natural experiment as the research study where the exposure to the event or intervention of interest has not been manipulated by the researcher.” (Craig et al. 2012).

#### Type of participants or population of interest

General or specific population studies examining PA in educational settings (university level and below), the workplace, in cities and neighborhoods, and older institutionalized adults or adults in neighborhoods or communities. Studies aimed at specific populations with diseases or conditions, such as neuromuscular diseases (multiple sclerosis, cerebrovascular disease, muscular dystrophies), musculoskeletal diseases (lupus, arthritis, osteoarthritis) or cardiovascular diseases (myocardial infarction, cardiac arrhythmias, valvular heart diseases, etc.) were excluded. Studies on athletes were also excluded.

#### Type of interventions

Studies that evaluated the promotion of PA from the perspective of structural- or physical-level environmental modification such as the construction of parks, bicycle paths, cycle lanes or modifications in cities were included.

#### Type of outcomes

The main outcome was PA in terms of variation or levels measured objectively or subjectively. At the objective level, measurements included pedometers, accelerometers, heart rate monitors and direct and indirect calorimetry. Subjective measurements included self-reports or questionnaires like the International Physical Activity Questionnaire (IPAQ) System outlines expected behavior for students in each activity throughout the daily (CHAMPS) or the Physical Activity Recall, among others. Both the objective and subjective measurements could be expressed continuously [such as total energy expenditure (Kcal/Kg/week, kcal/week), metabolic consumption in METS, oxygen consumption or differences in respiratory exchange ratio (CO_2_/*V*O_2_), heart rate, variability in heart rate, total minutes of PA or number of steps, among others] or categorically (such as of light, moderate or vigorous PA). Participation in the programs, percentage of physical activities performed, amount of PA performed, measures of fitness level if they were reported in metabolic expenditure or oxygen consumption, and measurement scales of an individual or group PA were also included.

### Search strategy

To identify the studies, a search was carried out up to January 2022 in electronic databases: Medline/Pubmed, Web of Science, Science Direct and CINAHL, using Mesh, Decs and Emtree terms. Supplementary material 1 provides the search strategies. Additionally, a search of crossed references was done manually as well as a search of gray literature in specialist journals, university repositories or general websites related to the topic.

### Study selection

For the selection, two reviewers (EH, EC) screened the records by title and abstract according to the inclusion criteria. The selected studies were then blinded, read in full-text format by both reviewers and their results and conclusions compared. In the case of disagreements, a third reviewer acted as a peer evaluator to enable a definitive conclusion and to settle such disagreements. To optimize the work at this stage, the Rayyan© software (Elmagarmid et al. 2014) was used.

### Data extraction and risk of bias evaluation

For the data extraction, a spreadsheet was created, in which the characteristics of the studies were recorded and included the title, authors, year and place of publication, program undertaken and its characteristics, start and end date of the program, scope of intervention, outcomes and measures considered, and reported results.

The risk of bias was assessed using the recommendations and evaluation criteria of the ROBINS I tool for non-randomized studies of interventions (Bero et al. 2018). This made it possible to evaluate specific risks of bias at three points in the study: point 1 or pre-intervention, where the biases of confounding and participant selection were considered; point 2 or during the intervention, where measurement bias was assessed; and point 3 or post-intervention, where the biases in the interventions performed, outcome measurement and attrition bias were considered. Each risk of bias domain was graded by considering the criteria as described by the ROBINS I tool and evaluated qualitatively. The risk of bias level was not evaluated quantitatively, since the ROBINS 1 tool is designed for observational studies, but it provided an assessment of the risk of bias that is often associated with natural experiments. When the risk of bias was not apparent in a given study, a third evaluator assisted with the assessment of the risk of bias.

### Data analysis

The data analysis was undertaken qualitatively through figures and tables that show the information revealed in the data extraction process. This way, the scope of the intervention programs, methodological findings and results of each study could be visualized in an organized manner conducted according to Cochrane Collaboration (Deeks et al. 2011).

## Results

**Fig. 1:**
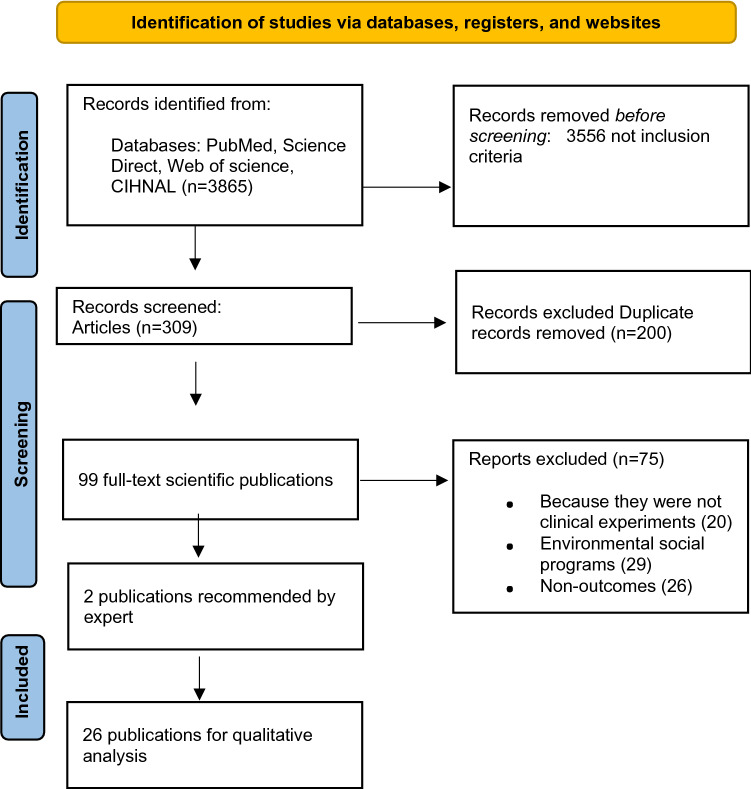
Selection process Prisma flow chart

In total, 3865 articles were found in the 4 interrogated databases. After eliminating the duplicates (200), two evaluators screened 3665 titles and abstracts and excluded 3566 for not fulfilling the inclusion criteria. Thus, 99 full texts were reviewed in depth to determine that 24 studies fulfilled the eligibility criteria and 2 new studies were included for the expert’s recommendation. The reasons for exclusion were: 20 articles did not report on natural experiments, 26 did not have outcomes of interest, and 29 reported on natural experiments but focused on social programs with no structural or physical environmental modification. It is important to note that, of the 26 studies included, 9 worked jointly on physical environmental modifications and social programs. The selection process is presented in Fig. 1.

### Characteristics of the studies included

**Fig. 2:**
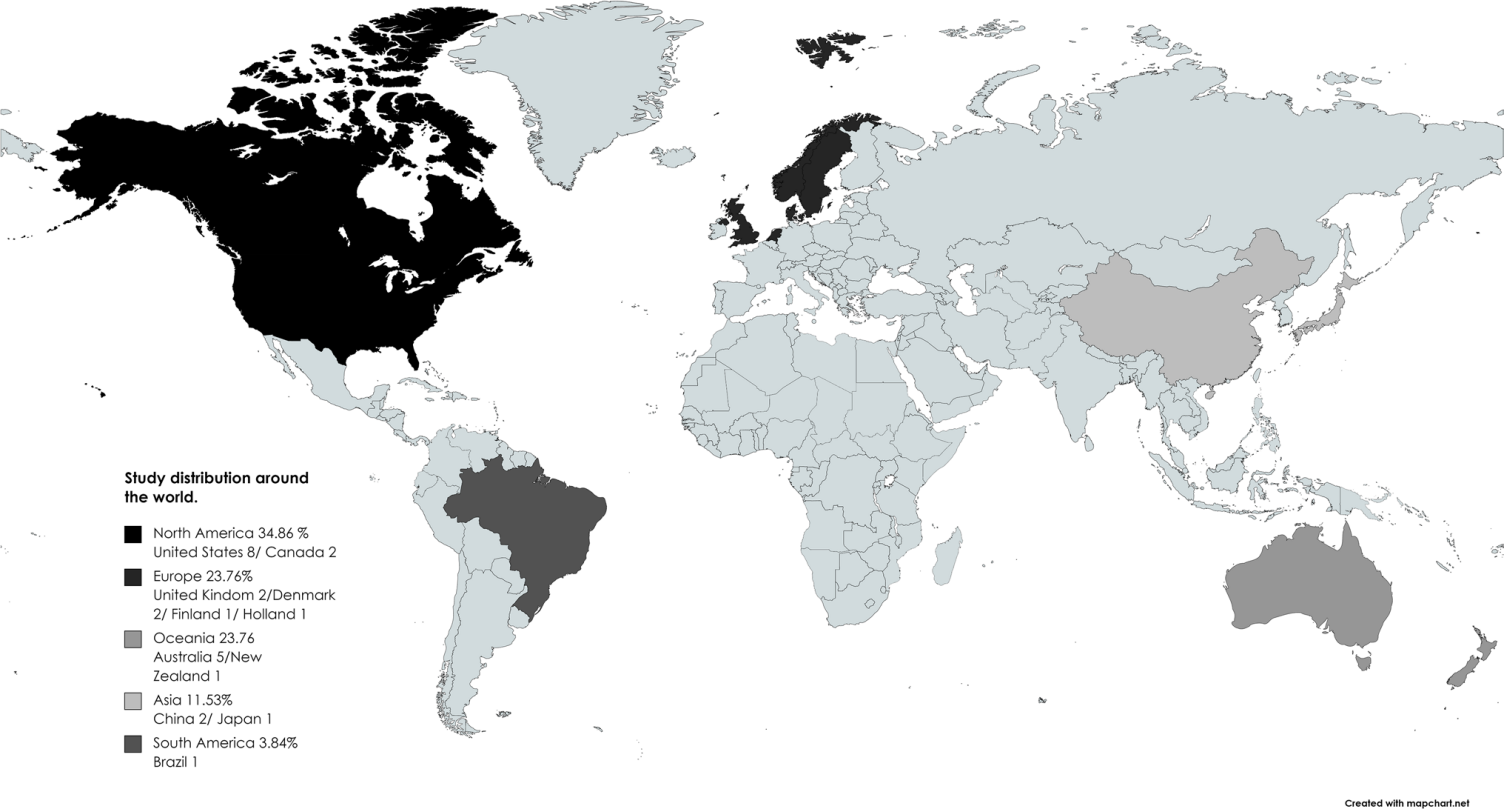
Study distribution around the world

The 26 studies included (Schultz et al. 2017; Dill et al. 2014; Auchincloss et al. 2019; McCormack et al. 2016; Ogilvie et al. 2016; Simões et al. 2017; Lee et al. 2017; Cohen et al. 2012, 2019; Jindo et al. 2020; Cranney et al. 2016; Brittin et al. 2017; Andersen et al. 2017; Zhu et al. 2018; Sun et al. 2020; Mölenberg et al. 2019a; Fitzhugh et al. 2010; Veitch et al. 2012; Aittasalo et al. 2019; Hooper et al. 2018; Quigg et al. 2012; Giles-Corti et al. 2013; McGavock et al. 2019; Demant Klinker et al. 2015; Ward Thompson et al. 2013; Jancey et al. 2016) were natural experiments published between 2010 and 2020. These studies were developed in different parts of the world (Fig. 2). The studies examined interventions from different perspectives including structural modifications for access and the use of parks and green areas (8 studies), installation of fitness areas or playground equipment in parks (2 studies), construction of new active transportation alternatives such as bicycle lanes or other transportation systems (3 studies), modification of cities, urban spaces or neighborhoods (7 studies), modification of school environments (2 studies) and work spaces (4 studies). The general characteristics of each study are shown in Table 1.

**Table 1:** Characteristics of the studies included and areas of emphasis

Authors/year	Country/Continent	Infrastructural focus	Intervention program	Description of the group
Schultz et al. (2017)	United States/North America	Parks and surrounding	A crossing area was built to access in the parks, PA was measured prior to construction 2012, post-construction 2013, and in 2014	Ten natural experiments focused on parks interventions or installation of exercise areas in them Although these interventions were designed in different latitudes in the world, they converge in a common point: modification of surroundings, infrastructure or equipment in green parks or spaces The primary goal to increase and promote physical activity in the population by remodeling or building in natural spaces, to obtain a greater facility to practice physical activity and increase activity levels
Auchincloss, et al. (2019)	United States/North America	Parks and surrounding	Construction of a 1-km greenway in an economically disadvantaged area	
McCormack et al. (2016)	Canada /North America	Parks and surrounding	Special areas for dog walking were built in parks to promote PA among the users	
Cohen et al. (2012)	United States/North America	Parks and surrounding	Fitness areas were installed in the parks in the county and city of Los Angeles, California. Use was estimated before the manufacture of the areas and after their adaptation in 12 parks	
Cohen et al. (2019)	United States/North America	Parks and surrounding	Use of six San Francisco neighbourhood parks and park-based physical activity levels over a 6-year period, during which five of the six parks were renovated	
Cranney et al. (2016)	Australia/Oceania	Parks and surrounding	An outdoor gym was installed in a park in Australia to promote the activity in older adults	
Veitch et al. (2012)	Australia/Oceania	Parks and surrounding	A neighborhood park in low-income areas of Victoria was fenced and remodeled in its areas for exercise and PA	
Quigg et al. (2012)	New Zealand/Oceania	Parks and surrounding	Modification of community play areas in two park areas in two low-income neighborhoods. Correlated with a control group from other unimproved play areas	
McGavock et al. (2019)	Canada /North America	Parks and surrounding	Impact of a frozen pathway on the users’ visits to estimate physical activity pattern associated with the pathway in the winter in Canada	
Ward Thompson et al. (2013)	United Kingdom/Europe	Parks and surrounding	Economically deprived park area modified and remodeled from the areas of trees and footpaths to create greater attraction to the public and increase PA	
Dill et al. (2014)	United States/North America	Active transport	Bicycle paths were planned and built and compared to the control	Three studies focused on the modification of the physical space to facilitate and implement active transport. This is understood from the construction of walking spaces, to the adaptation of bicycle lanes or exchange of transport between bus and walking. The three natural experiments concentrate on the commute of transport to promote physical activity
Ogilvie et al. (2016)	United Kingdom/Europe	Active transport	A new system of transport was built in Cambridge between 2007 and 2011, with 19 km of road for buses and bike or walking lanes	
Sun et al. (2020)	China/Asia	Active transport	A new metro line with 24 stations in a population that had no metro line and the change of habits in the type of transport was determined	
Simões et al. (2017)	Brazil/South America	Active cities, urban modification	Academy program of city of Pernambuco Brazil with 184 cities that participated in the activity in three groups	Seven studies focused on a modification of cities, neighborhoods and urban areas, with urban geoplanning for the design of neighborhoods and their modification that allowed walking and the practice of physical activity in architectonically planned spaces prior to their construction. All with the aim of favoring physical activity in times of relaxation, leisure and recreation
Andersen et al. (2017)	Denmark/Europe	Active cities, urban modification	Urban renovation of an economically depressed area in Denmark, in which parks and areas for physical activity were built	
Mölenberg et al. (2019a, b)	Holland/Europe	Active cities, urban modification	18 new spaces in economically depressed sectors, in which spaces for the promotion of PA were created in Rotterdam	
Hooper et al. (2018)	Australia/Oceania	Active cities, urban modification	RESIDE Project in Perth, Australia, the neighborhood was modified and built according to urban planning norms with green spaces and parks nearby	
Fitzhugh et al. (2010)	United States/North America	Active cities, urban modification	An urban greenway/trail was retrofitted in a neighborhood that lacked connectivity of the residential pedestrian infrastructure, pre and post measurements	
Giles-Corti et al. (2013)	Australia/Oceania	Active cities, urban modification	Effect of relocation in neighborhoods for transport walking and recreational activity among neighborhood residents	
Demant Klinker et al. (2015)	Denmark/Europe	Active cities, urban modification	Changes were made in a low-income neighborhood and children from public schools were invited to participate in the activities in the surroundings	
Lee et al. (2017)	United States/North America	School infrastructure	Transfer of a group of students at a school outside a neighborhood to one with the school in the neighborhood and to see the change in behavior from sedentary to active and to the transport	Two studies focused on the modification of school structure or to the inside or outside of the school, to promote physical activity
Brittin et al. (2017)	United States/North America	School infrastructure	A school was designed by architects and educators with modifications and adaptations that promote physical activity, spacious halls with chairs and tables adapted to allow free circulation in the environment, the students are transferred from an old school to a new one	
Jindo et al. (2020)	Japan/Asia	Work environment	Three offices were renewed with height-adjustable desks and spaces that allow for greater activity with the projection of well-being and fitness	Four natural experiments focused on modifications of the work environment and restructuring of the building, office and workstation, with adjustment of the spaces or the modification of active transport behaviors in the use of walking, cycling or taking the bus instead of the car as means of transport in the work environment
Zhu et al. (2018)	China/Asia	Work environment	PA and sedentary behavior among the employees of a company in response to job modification, it is a two-arm experiment, one of intervention with modification of the job, adaptation of the chairs and desks	
Aittasalo et al. (2019)	Finland/Europe	Work environment Active transport	Two-arm natural experiment with two groups of companies in a two-phase socioecological model to determine the use of the bicycle or walking as a means of active transport	
Jancey et al. (2016)	Australia/Oceania	Work environment	A work office was remodeled in its structure inside the building, changing its distribution and leaving more spaces for free circulation and activity	

### Measurements of the levels of physical activity in the studies

All outcome measures have previously published evidence of validity and reliability globally and while not all measures are used in every study, some of the same measurement tools were used in more than one study. Instrumental measures were identified as the accelerometer in 8 studies (Dill et al. 2014; Ogilvie et al. 2016; Jindo et al. 2020; Brittin et al. 2017; Andersen et al. 2017; Quigg et al. 2012; Demant Klinker et al. 2015; Jancey et al. 2016), pedometer in 1(McGavock et al. 2019), and satellite location and data processing systems like Global Positioning System (GPS), Active Physical Activity Location Measurement System (ACTIVE PAL 3C), Physical activity location measurement system (PALMS) in 4 studies (Dill et al. 2014; Andersen et al. 2017; Zhu et al. 2018; Demant Klinker et al. 2015). Non-instrumental measures recognized worldwide such as the System to Assess the Practice of Physical Activity and Recreation in Parks and Natural Surroundings (SOPARC) were identified in 6 studies (Schultz et al. 2017; Auchincloss et al. 2019; Cohen et al. 2012, 2019; Cranney et al. 2016; Veitch et al. 2012), the IPAQ, a PA questionnaire, in 2 studies (Dill et al. 2014; Simões et al. 2017), and the Recreation Physical Activity Questionnaire (RPARQ), in 1 study (Ogilvie et al. 2016).

It should be mentioned that 20 of the studies reported measuring PA levels with scales of light, moderate or vigorous. In some, the application of general surveys of perception of activity and its level or walking was reported, up to counting the activities performed in each of the protocols. (See Table 2).

**Table 2:** Main methodological characteristics of the studies

Authors/year	Population	Groups	Statistical analysis	Physical activity measurements
Schultz et al. (2017)	476 neighborhoods with parks, 2276 adult and child participants	Pre- and post-intervention at passageway to parks	One-way and two-way ANCOVA	SOPARC LPA, MPA, MVPA, VPA of physical activity, Metabolic expenditure in METS
Dill et al. (2014)	8 bicycle lanes and 11 control streets, 353 adults	Pre- and post-intervention with two groups in bicycle lanes	Multinomial logit model, negative binomial logit, linear regression	IPAQ Accelerometers MVPA, of activity Location with GPS
Auchincloss et al. (2019)	8783 participants, 175 trips, 40,000 users	1-km green corridor compared to control group	Adjustment for differences, hierarchical logistic model	SOPARC LPA, MPA, MVPA, VPA
McCormack et al. (2016)	400–800 users in 4 parks	Two intervention and two control parks	Chi-square, Pearson’s *z* test, Bonferroni correction. Binary logistic regression stratified by park with odds ratio	LPA, MPA, MVPA, VPA of physical activity Metabolic expenditure in METS
Ogilvie et al. (2016)	1164 individuals, both genders	New transport system in Cambridge 19 km of road for buses and bicycle or walking lanes	Multivariate regression. Intention-to-treat analysis with a per-protocol analysis in a clinical trial. Qualitative analysis as per social theory	RPAQ Accelerometers Moderate-vigorous level of activity Location with GPS
Simões et al. (2017)	8900 users in 84 cities in Pernambuco	Two intervention groups with modification and physical activity programs and control without modification	Regression with a stability model for the reclassification of the city, Logistic and Glimmix for raw and adjusted odds ratios	IPAQ leisure and transport Level of activity according to walking and participation
Lee et al. (2017)	165 surveys of students were processed	Two intervention groups with modification of a neighborhood school	Imputation model of variables, binary logistic regression in three adjusted models	Survey of forms of transport to and from school in children who were transferred and level of activity
Cohen et al. (2012)	23,577 users in 12 parks	Two groups with fitness areas were installed in parks in the county vs. control park	Linear regression model, weight adjustments, propensity score and weighted regressions and mixed models	SOPARC LPA, MPA, MVPA, VPA Metabolic expenditure in METS
Cohen et al. (2019)	Six parks and the population ranged from 9735 to 45,714 residents	Five intervention and one control parks	Difference-in-differences approach in estimating short-term and long-term effects differences among study arms and secular trends in outcomes	SOPARC Metabolic equivalents (METs)
Jindo et al. (2020)	42 participants 39 controls and 13 renovations	Two intervention groups with renovation of offices with the adoption of active bas workstation and control office	Descriptive statistics with analysis of means and standard deviation Analysis of two-way repeated measures	Accelerometers MVPA, of activity Video cameras
Cranney et al. (2016)	23,905 users in the parks	An outdoor gym was installed in a park in Australia pre- and post-measurement	Descriptive statistics with z-tests to determine differences, chi-square for comparison the medias and reliability tests	SOPARC LPA, MPA, MVPA, VPA
Brittin et al. (2017)	32/20 participants from schools in Virginia and New York	A school was designed by architects and educators with modifications was compared to a control	Difference-in-differences model and mixed linear method with control of variables for gender, and binary for race	Accelerometers MVPA, of activity
Andersen. et al. (2017)	354 adolescents pre- renovation and 319 post	Urban renovation study in an economically depressed area in Denmark pre- and post-measurement	Descriptive statistics with t- and Wilcoxon tests, multilevel analysis to detect differences adjusted for variables	Accelerometers MVPA, of activity Location with GPS PALMS activity system
Zhu et al. (2018)	52 participants in the study and 36 in the post-test, 12 test control and 24 intervention	Two groups. The intervention was called stand up and move and a new adjustable workstation was provided compared to a control	Descriptive statistics with tests to detect differences between the groups, measurement of effect of the activity was determined according to Cohen’s model	ActivePAL3C to measure the activity, Posture Time MVPA level of activity
Sun et al. (2020)	Number of trips and change in the types of trips	An intervention group with a new subway line with 24 stations pre and post measurement	*t*-tests and difference-in-differences between the groups	Questionnaire of the preferences and type of uses of transport, bus, bicycle, walking or car, how long and with what frequency
Mölenberg et al. (2019a, b)	*n* = 1841 ages 6 (2008–2012) and 10 (2012–2015) (*n* = 1607) outside playground (*n* = 1545). Sedentary behavior	Two intervention groups with 18 new spaces in economically depressed areas compared to a control	Descriptive statistics with tests to detect mixed differences and linear models	Distance from the houses and neighborhoods to sporting grounds Use of spaces Hours spent on activity in open environments during the week and at the weekend Level of physical activity of the participants
Veitch et al. (2012)	Number of participation counts of users in parks (1350)	A park in neighborhood in low-income areas of Victoria was fenced and remodeled compared to a control	Two-way ANOVA and test to determine differences between the times of the groups	SOPARC LPA, MPA, MVPA, VPA level of physical activity
Fitzhugh et al. (2010)	Number of participations counts of users in trail	Pre- and post-intervention at trail or greenway	Analysis used Fisher’s exact tests to detect statistically meaningful relationships among the counts	Number of counts of the use of the trail Number of pedestrian, walking and cycling
Aittasalo et al. (2019)	44 companies, 1833 workers	11 companies. The presence of lanes or roads for cycling or walking and use by workers for active transport was determined compared to control	Differences between the times of the groups, Mann–Whitney *U* test and Wilcoxon test to detect differences	Questionnaires on the use of the bicycle or walking as a method of active transport Time of use in hours or minutes and number of times per week on the activity LPA, MPA, MVPA, VPA level of physical activity
Hooper et al. (2018)	1813 users and 6 measurements areas	Modification and construction of a neighborhood according to city-planning regulations with green spaces and near parks pre and post measurement	Logistic regression model adjusted to multiple level and determination of Odds ratio	Walking as recreation is measured Level of physical activity Questionnaire on physical activity in the neighborhood Measured the frequency and level of activity in walking Level of activity and calculation of MVPA
Quigg et al. (2012)	156 children	Playgrounds in 6 local parks were modified and compared to a control	Linear mixed model	Accelerometers MVPA level of activity Physical activity questionnaire
Giles-Corti et al. (2013)	1420 participants in the study	Modification and construction of a neighborhood according to city-planning regulations with green spaces and near parks pre- and post-measurement	Linear mixed model, adjusted multiple regression model	Walking as recreation is measured Level of physical activity Effects of neighborhood change on walking and bicycle use
McGavock et al. (2019)	176 users	Two intervention groups of two frozen waterways in winter	Model *t* test and multivariate regression	Number of counts of the use of the tracks in the groups by means of an infrared system Level of physical activity in users who attended the track with the use of pedometers on their waist Moderate-vigorous activity of the participants and counts steps
Demant Klinker et al. (2015)	367 children in 3 public schools	Changes in a low-income neighborhood and children from public schools were invited to participate in the activities	Descriptive statistics with tests to detect mixed differences, chi-square and *t* test	Accelerometers MVPA level of activity Frequency and level of activity of walking or participation in activities
Ward Thompson et al. (2013)	215 users in the area	Two groups in an economically deprived park area which is modified and remodeled compared to a control	Mann–Whitney *U* test to detect differences between the groups	Frequency of activity, neighborhood and wooded area characteristics Surrounding environment and the frequency with which they were leaving this area Frequency of physical activity and attitudes to it
Jancey et al. (2016)	42 participants in the study	A work office was remodeled in its internal structure of the building pre and post measurement	*t* tests and difference in means between the groups	Accelerometers Number of activity counts or time spent sitting MVPA level of activity

Main methodological

*LPA* light physical activity, *MPA* moderate physical activity, *MVPA* moderate-vigorous physical activity, *VPA* vigorous physical activity, *METS* metabolic equivalents, *SOPARC* system to assess the practice of physical activity and recreation in parks and natural surroundings, *IPAQ* physical activity questionnaire, *RPARQ* recreation physical activity questionnaire, *GPS* Global Positioning System, *PALMS* Physical activity location measurement system, ActivePAL3C active Physical activity location measurement system

The reports of PA were also expressed by counting the number of subjects participating in the programs, the levels of PA in percentage, minutes or total time, and the number of steps or metabolic expenditure. In addition, there were reports on changes in PA patterns, either in the use of transportation, management of the activity or performance. Some studies reported on the time spent sitting as a significant measurement to define PA.

### Effectiveness of the structural modifications

The outcome of PA was presented in the 26 studies (Schultz et al. 2017; Dill et al. 2014; Auchincloss et al. 2019; McCormack et al. 2016; Ogilvie et al. 2016; Simões et al. 2017; Lee et al. 2017; Cohen et al. 2012, 2019; Jindo et al. 2020; Cranney et al. 2016; Brittin et al. 2017; Andersen et al. 2017; Zhu et al. 2018; Sun et al. 2020; Mölenberg et al. 2019a; Fitzhugh et al. 2010; Veitch et al. 2012; Aittasalo et al. 2019; Hooper et al. 2018; Quigg et al. 2012; Giles-Corti et al. 2013; McGavock et al. 2019; Demant Klinker et al. 2015; Ward Thompson et al. 2013; Jancey et al. 2016) shown in Table 3 in which the effectiveness of the programs was variable. The level of PA was greater in some structural-level environmental modifications than in others while no difference between intervention and control groups were observed. Table 3 shows the participation in the activity and global measures time of the activity in minutes, percentage increase or decrease in PA.

**Table 3:** Effectiveness of the structural modifications

Authors	Activity level					Sedentary time
	Counts	% physical activity	Time physical activity	Steps	Metabolic work	
Schultz et al. (2017)	↑ from 2080 to 2276 users	_	↑ 889–921 MPA and from 81 to 122 VPA time	–	↓ 4613–4014 METS post	–
Dill et al. (2014)	↓ 307–240 and from 183 to 123 counts of bicycle users in second follow-up	↓ MVPA from 39.5 to 39.4%	↓ Total time on bicycle from 104 to 66 min and walking from 107 to 89	–	–	–
Auchincloss et al. (2019)	↑ 100–116 users	↑ MPA by 45% with and Odds 1.45 CI 1.06, 1.98 riding bicycle, running or fast walking	–	–	–	–
McCormack et al. (2016)	↑ 351–658 in users (41.3 vs. 56.5%)	No changes in % between the parks	Proportion of walkers (58.0–68.3%) and cyclists (25.9–15.9%) in time of activity	–	–	–
Ogilvie et al. (2016)	↑ 1143–1710 adults participating	16% of the achieved daily MVPA in this population and about 30% of the recommended minimum level of MVPA	↑ Total time of physical activity in 55 min of MVPA given the commute < 150 min 6.5 min MVA daily, > 150 min 14.3 min of daily activity and MVPA	–	↑ 0.7 METS in people using the bus, between 0.6 and 1.6 METs using the car and walking or cycling 2.5 and 3.9 METs	–
Simões et al. (2017)	↑ 10,000 participants in all the study	The proportion of individuals that reached the LPA guidelines was 25.8%	Increase in the time of participation for those who never participated and began their participation and to reach the levels (or = 1.61; 95% CI 1.18; 2.20, less than 6 months 1.83; 95% CI 1.17; 2.86, *p* value = 0.0078 more than 6 months (or = 5.06; 95% CI 3.34; 7.67, *p* value b0.0001)	–	–	–
Lee et al. (2017)	Out of 165 subjects 68 changed to active transport	41% active transport by bicycle or walking, 58.8% no change	–	–	–	–
Cohen et al. (2012)	↑ 442–952 subjects in the parks	↑ 66–72% MVPA	↑ Number of time sessions and number of exercise sessions 2.76 vs. 2.4 in each group	–	↑ 16,900–19,800 and 18,234 METS after first and second follow-up	–
Cohen et al. (2019)	↑ 368 users of playground areas in the five parks ↑ 1226 users in 2015	–	–	–	↑ 70% in MET-hours in all renovated parks per observation ↑ MET-hours total park use and total 480 and 636%, respectively	–
Jindo et al. (2020)	–	–	Total PA, min/working hours 155 ± 42 173.2 ± 28.6 0.164 Light-intensity PA, min/working hours 122.3 ± 36.4 130.4 ± 27.1 0.475 Moderate- to vigorous-intensity PA, min/working hours 32.7 ± 15.7 42.8 ± 15.9 0.063	–	–	↓ Sedentary B 346.8 ± 28.6 to 321.2 ± 17.8 min/working-hours
Cranney et al. (2016)	↑ 204–428 gym area users	↑ 6–40% MVPA in gym use and 19–28% in children and 6–8.6% adults	–	–	–	–
Brittin et al. (2017)	–	–	↑ LPA from 140 to 150 min, with total increase of 67 min and ↓ MVA from 20 to 10 min	–	↑ 152.3 LPA and decrease of -61 in MVPA	↓ 81.2 min/day from 270 to 245
Andersen. et al. (2017)	354 users pre- and 317 post-renovation	–	↑ In minutes of activity from 44.8 to 99, from 12.3 to 28 LPA and 1.5 to 3.5 MVPA	–	–	–
Zhu et al. (2018)	–	↑ 24.9–27.5 LPA and ↓ 6.6–6.5 MPA	–	–	–	↓ 337–281 sitting and ↑ 111–165 sedentary time
Sun et al. (2020)	↓ 5436–1770 participants	↓ % of time journeys for work and not walking bicycle and bus between 2 and 28% in each, and increase in metro, car and metro to walking from 28 to 33%	–	–	–	–
Mölenberg et al. (2019a, b)	171 children participated in the use of 600 m of new spaces	Having 600 m of space dedicated for PA no % change in outdoor play PA in children 6–10 years compared to control	Children aged 10 years played 40 min more and in families with low maternal education level the children played 96 min more during the week	–	–	Reducing the distance to 100 m did not present effects in sedentary behavior or increase in activity
Veitch et al. (2012)	↑ 235–985 users time 2	↑ 16–26% MVPA from 38 to 257 subjects in MVPA	↑ 155–368 subjects in walking time increasing the time	–	–	–
Fitzhugh et al. (2010)	↑ with a median increase of 8 counts PA experimental 2-h count of total physical activity walking and cycling was significantly (*p* = 0.028) higher in the experimental neighborhood	–	–	–	–	–
Aittasalo et al. (2019)	↑ 646–1013 cycling and 309–346 walking	↑ commute via bicycle 36% and walking 11% increasing of PA	↑ commute time from walking and cycling for transport	–	–	–
Hooper et al. (2018)	1777 participants live in areas of modification	54% report recreational walking in the area, 44% MVPA	Living in an area of 400 m probability 3 times > of doing any type of MVPA in the neighborhood (OR = 3.17, 1.02–9.81) Access to a local park in a radius of 400 m was associated with 22% PA (OR = 1.219, 1.018–1.460) of doing any type of physical exercise in the neighborhood	–	–	–
Giles-Corti et al. (2013)	↑ 388–1420 users time 1 to time 2	↑ 15–21.1 min min/week in recreational walking and ↓ 8.5 min walking as transportation	–	–	–	–
McGavock et al. (2019)	↑ 405–1813 and 2449–4516 counts per day in two follow-ups	–	↑ MVPA in minutes (32 vs. 25 min) and accumulated 27 ± 18 min of MVPA	4195 steps in 39 min, 4796 vs. 3987 steps during the week	–	
Demant Klinker et al. (2015)	Total users 367 during the week and 176 weekends	↑ % of children in active transport 91%, in school playgrounds 98% or green spaces during the week in contrast to weekend where they are more in sport clubs and at home	school domain (302.3 min), leisure (247.1 min), home (206.6 min) transport (47.2 min) during week and weekends leisure (337.3 min), home 308.6 min) transport (63.8 min)	–	–	–
Ward Thompson et al. (2013)	↑ 7–34 visitors in the area	↑ 25% of activity greater than 5 h of activity	Greater visiting time to modified woods	–	–	–
Jancey et al. (2016)	–	↑ 11–17% LPA and ↓ 3.8–3.2 MPA and 0.07–0.06 VPA	↑ 35–57 min LPA and 36–19 MPA and 49–60 min VPA	↑ 26–63.60 max, per minute 6.82–10.28 and per day 3238–4924	–	↓ 84–79% sitting and time min 401–381

Effectiveness of the programs

*LPA* light physical activity, *MPA* moderate physical activity, *MVPA* moderate-vigorous physical activity, *VPA* vigorous physical activity, *METS* metabolic equivalents

### Activity level

Seventeen studies (Schultz et al. 2017; Dill et al. 2014; McCormack et al. 2016; Ogilvie et al. 2016; Simões et al. 2017; Cohen et al. 2012; Jindo et al. 2020; Brittin et al. 2017; Andersen et al. 2017; Mölenberg et al. 2019a; Veitch et al. 2012; Aittasalo et al. 2019; Hooper et al. 2018; McGavock et al. 2019; Demant Klinker et al. 2015; Ward Thompson et al. 2013; Jancey et al. 2016) reported an increase in the PA level post-structural modification. For example, an increase in total activity time, varying between 10 and 30 min, a 6–28% increase and an increase of between 38 and 369 users participating in the activity were found between the pre- and post-structural modifications. The measurement of PA in the area of parks, especially in those with outdoor gyms included, were more effective at improving PA. It is important to emphasize that these changes were dependent on factors that were both internal and external to the practice of activity, such as the physical environment, sex, socioeconomic level of the participant, and personal characteristics.

The intensity, or level, of PA was often described as light, moderate, or vigorous levels were used as a measure of PA in these studies. However, although the use of light PA was consistent throughout all studies, some studies further divided PA and reported the intensity of PA as moderate, moderate-vigorous, and vigorous levels while other studies combined them into one level. At the specific intensity of PA, the duration of light PA increased between 22 and 67 min or between 6 and 8% while the duration of moderate PA increased from 1 to 43%. The moderate-vigorous level of PA increased from 6 to 36%, but in 3 studies no change in the level of PA was observed (Dill et al. 2014; McCormack et al. 2016; Lee et al. 2017). At the vigorous level, no increase in PA was reported in 80% of the studies while the remaining 20% reported only a slight increase of 5–7%. A major difference between the studies is that those in the area of work and transportation report greater increases in light and moderate activity. Finally, in one of the largest studies conducted on urban modification, RESIDE in Australia, a 54% post-renovation increase in minutes/week of recreational walking was reported along with a reduction of minutes walking in active transport, suggesting participants preferred recreational walking over active transport walking when both options are available. Similarly, an increase in moderate-vigorous activity was observed in this population, with an Odds Ratio 3.17, (I.C 1.02-9.81) demonstrating the effect of activity in the area of the new neighborhood which is summarized in Table 3.

### Counts

Only 9 studies reported participation counts which were defined as the number of individuals using a new structural-level environmental modification, of which 8 described modifications of parks (Schultz et al. 2017; Auchincloss et al. 2019; Cohen et al. 2019; McCormack et al. 2016; Veitch et al. 2012; Quigg et al. 2012; McGavock et al. 2019; Ward Thompson et al. 2013) and 1 in cities (Fitzhugh et al. 2010) with all studies showing an increase in the number of subjects using the area. In contrast, the 1 study of active transport in bicycle lanes (Dill et al. 2014), showed a decrease in post-participation measurement as shown in Table 3. Regarding the increased number of participants in the studies examining the modification of parks, there was a greater increase in participant use when the structural modification included outdoor gyms while there was less of an increase observed when the parks were modified only for recreation and exercise purposes (Veitch et al. 2012).

One study described the construction of a green area in the city (Auchincloss et al. 2019) and found a 45% increase in walking, cycling, and running in the park post-remodeling with an odds ratio (OR) of 1.46 (CI 1.06–1.98). In contrast, a study examining the remodeling of areas in parks for users with pets found an increase in the number of subjects in the remodeled parks increased, but without a significant increase in the PA of those subjects (McCormack et al. 2016).

### Metabolic consumption and number of steps

The third and fourth outcomes are metabolic consumption in METS and the time spent sitting versus active time. METS were reported by 5 studies (Schultz et al. 2017; Ogilvie et al. 2016; Cohen et al. 2012, 2019; Brittin et al. 2017), of which 3 were in parks, 1 was in schools, and 1 in the area of transportation. Fourth of the 5 studies found an increase in metabolic work (Ogilvie et al. 2016; Cohen et al. 2012, 2019; Brittin et al. 2017) and 1 found a decrease (Schultz et al. 2017) as shown in Table 3. The time spent sitting versus active time was reported in 4 studies, with 3 in the workplace (Jindo et al. 2020; Zhu et al. 2018; Jancey et al. 2016) and 1 in a school (Brittin et al. 2017) and all of the studies found not only an increase in PA but less time spent sitting in both the work and school populations. Furthermore, in the workplace studies, as shown in Table 4, there was a reduction in sitting time and of seated work, with an increase in both standing work and light PA. In fact, light PA increased from 130 to 150 min while no increase in moderate-vigorous PA was observed in the work areas. Similarly, in schools there was a reduction in sitting time during the students’ day, although no increase in light PA was reported (Table 3). It is worth noting that these 4 studies are more controlled given that they are in closed areas like a company or a school, which enables a direct and accurate follow-up of the above outcomes.

The outcome, number of steps, was reported in 2 studies using pedometers or accelerometers with (McGavock et al. 2019; Jancey et al. 2016), 1 study examining modifications in parks with frozen waterways and the other study examining the effect of modifications to the workplace on PA. Both studies found an increase in moderate-vigorous activity with a greater number of measured steps related to the use of frozen waterways and to the use of the space for physical activity in the workplace as shown in Table 4.

### City-wide changes

Seventh studies examined the impact of structural modifications in cities (Simões et al. 2017; Andersen et al. 2017; Hooper et al. 2018; Fitzhugh et al. 2010; Mölenberg et al. 2019a; Giles-Corti et al. 2013; Demant Klinker et al. 2015), with several favorable results on the increase in PA. For example, in the modification of 184 cities in Brazil it was found that the population of each city reached the light activity goals previously set forth in the project. In particular, increased light-PA was observed in men with a higher level of education yielding an OR of 1.46 (CI from 1.11 to 1.92). Furthermore, the study found that programs lasting longer than 6 months increased the OR for PA as shown in Table 3 (Simões et al. 2017). Fourth related studies on urban renovation described the modification of spaces in cities to increase PA particularly aimed at the populations of young people and children (Mölenberg et al. 2019a; Andersen et al. 2017; Fitzhugh et al. 2010; Demant Klinker et al. 2015) and found an increase in PA post-renovation, with greater activity in minutes/week for both active transport and use of green areas, especially in sporting scenarios and playgrounds.

In these studies, an increase in moderate-vigorous PA was reported, although the increase was not significant, post-renovation. However, greater attendance time with an average of 24 min was observed in the modified spaces. Greater light PA was found globally reflected by an increase in the minutes in the populations attending the activity.

Furthermore, urban modification studies in Denmark led to an average increase in PA per week of 7.8 min for light activity, 4.5 min for moderate-vigorous, and 13.1 min less sitting time. Overall, an average increase in play time for students of over 40 min/week was observed, which was particularly pronounced for children with mothers having a lower level of education. Interestingly, there were no significant differences in the groups according to the distance from the new areas modified (Mölenberg et al. 2019a).

### Commuting

In regard to commuting via active transport, several studies report the use of alternative transport modifications, such as cycling and walking as well as the measurement of the commute (Dill et al. 2014; Lee et al. 2017; Sun et al. 2020; Aittasalo et al. 2019). For both school and workplace cycle programs, an increase in both the number of users and the number of trips by bicycle was noted, especially where the user lives 1 mile near the travel area.

In a study on the construction of a subway, an increase in active transport was measured by an increase in cycling time, for one work group, as well as an increase in the walking activities associated with taking the subway (Sun et al. 2020). One of the largest studies in the United Kingdom, where commuting changed from car to bicycle, reported an increase in minutes of bicycle use and a reduction in car use lead to an overall rise in commuting time from 10 to 150 min per week with a rate risk ratio RRR 2.5 CI 1.2–5.0. Additionally, greater METs were found for the use of the bicycle and walking in the moderate-vigorous activity and an increase of 150 min/week of walking was observed (Ogilvie et al. 2016), as shown in Table 3.

### Risk of bias in the included studies

Table 4 illustrates the results of the evaluation of the risk of bias in the studies included in this systematic review. Although the studies have different degrees of risk of bias, risk of bias is inherent in natural experiments. The observed biases are present in pre-intervention, during the intervention and post-intervention, due to access and follow-up of the population. However, where applicable, efforts were made to control for confounding variables.

**Table 4:** Risk of bias in the studies included

Phase	Bias	Evaluation
Pre-intervention	Confounding	Of the 26 studies, 5 have a previously published protocol (Anderssen et al. 2017; Ogilvie et al. 2016; Aittasalo et al. 2019; Giles-Corti et al. 2013; Hooper et al. 2018) that contrasts what was planned to what was implemented. The 19 remaining published the analysis a posteriori, which leads to the determination that there is bias in some measurements since what was reported is only after implementation In the 26 (Schultz et al. 2017; Dill et al. 2014; Auchincloss et al. 2019; McCormack et al. 2016; Ogilvie et al. 2016; Simões et al. 2017; Lee et al. 2017; Cohen et al. 2012, 2019; Jindo et al. 2020; Cranney et al. 2016; Brittin et al. 2017; Andersen et al. 2017; Zhu et al. 2018; Sun et al. 2020; Mölenberg et al. 2019a; Fitzhugh et al. 2010; Veitch et al. 2012; Aittasalo et al. 2019; Hooper et al. 2018; Quigg et al. 2012; Giles-Corti et al. 2013; McGavock et al. 2019; Demant Klinker et al. 2015; Ward Thompson et al. 2013; Jancey et al. 2016), it is clear there is a probability of confounding and selection bias since the interventions take place with a general selection of the population for participation in the studies rather than by previously specified criteria. In some, the criteria are more specific although in most they are very broad; thus, the risk of bias is higher in the studies conducted in outdoor environments like parks or programs in cities, since the inclusion of the subjects is given by the beginning of participation in the activity more than by previous selection of these subjects in the study. This is unlike those conducted in more controlled indoor environments such as companies or schools since the inclusion criteria can control more for confounding because it determines the admission of the participants or paired samples are managed
	Selection	12 (Ogilvie et al. 2016; Simões et al. 2017; Andersen et al. 2017; Zhu et al. 2018; Mölenberg et al. 2019a; Aittasalo et al. 2019; Hooper et al. 2018; Quigg et al. 2012; Giles-Corti et al. 2013; Demant Klinker et al. 2015; Ward Thompson et al. 2013; Jancey et al. 2016) of the 26 studies report inclusion criteria prior to participant selection, although the criteria are broad. Despite this, the characteristics to enter the study are defined, and are related to the characteristics of the reference population with a low possibility of selection biases. allow for less bias in the selection of the groups Of the 14 (Schultz et al. 2017; Dill et al. 2014; Auchincloss et al. 2019; McCormack et al. 2016; Lee et al. 2017; Cohen et al. 2012, 2019; Jindo et al. 2020; Cranney et al. 2016; Brittin et al. 2017; Sun et al. 2020; Fitzhugh et al. 2010; Veitch et al. 2012; McGavock et al. 2019),remaining, 9 have broad admission criteria, and select to the population by participation in the activity, for example going to a park and using the machines or the environment modified in cities, neighborhoods, bike lanes, this is more than for participation in the program. The remaining 5 (Cohen et al. 2012, 2019; Cranney et al. 2016; Veitch et al. 2012; McGavock et al. 2019) refer to selection of the population but do not specify how or the criteria to include them
During the intervention	Intervention measurement	In the 26 studies the interventions to apply are clear, in (Dill et al. 2014; Auchincloss et al. 2019; McCormack et al. 2016; Simões et al. 2017; Cohen et al. 2012; Cohen et al. 2019; Jindo et al. 2020; Zhu et al. 2018; Mölenberg et al. 2019a, b; Veitch et al. 2012; Aittasalo et al. 2019; Quigg et al. 2012; McGavock et al. 2019; Ward Thompson et al. 2013) of these there are two intervention groups and measurements are taken pre- and post-remodeling of the surroundings: parks, neighborhoods, cities, schools or working environment In 11 studies (Schultz et al. 2017; Ogilvie et al. 2016; Lee et al. 2017; Cranney et al. 2016; Andersen et al. 2017; Sun et al. 2020; Fitzhugh et al. 2010; Hooper et al. 2018; Giles-Corti et al. 2013; Demant Klinker et al. 2015; Jancey et al. 2016), there is no control group, they are pre- and post-remodeling studies of the area, with measurements taken twice or more The intervention protocols are explained in 5 of the studies (Anderssen et al. 2017; Ogilvie et al. 2016; Aittasalo et al. 2019; Giles-Corti et al. 2013; Hooper et al. 2018) with greater clarity since they have a protocol published prior to the execution of the study. In the other 21 studies, the interventions are explained in the definitive article, among the interventions found as seen in Table 1 21 are in open spaces (Schultz et al. 2017; Dill et al. 2014; Auchincloss et al. 2019; McCormack et al. 2016; Ogilvie et al. 2016; Simões et al. 2017; Cohen et al. 2012; Cohen et al. 2019; Cranney et al. 2016; Andersen et al. 2017; Sun et al. 2020; Mölenberg et al. 2019a, b; Fitzhugh et al. 2010; Veitch et al. 2012; Aittasalo et al. 2019; Hooper et al. 2018; Quigg et al. 2012; Giles-Corti et al. 2013; McGavock et al. 2019; Demant Klinker et al. 2015; Ward Thompson et al. 2013) with work in an outdoor environment such as parks, neighborhoods, cities, and 5 are in closed spaces (Lee et al. 2017; Jindo et al. 2020; Brittin et al. 2017; Zhu et al. 2018; Jancey et al. 2016) in an internal environment in companies and schools Regardless of the environments in which they are implemented, the work proposals are clear and specific for each of the studies included
Post-intervention	Interventions implemented	The 26 studies are clear in the interventions implemented according to what was posted in the methodology. In one of the studies, it is explained that the plan was to have a control group prior to the execution, but that measuring of the control group was not done during the implementation
	Outcome measures	The measures taken in the 26 studies are different from each other according to the environmental perspective Instrumental measures are found with the use of accelerometers, pedometers, activity follow-up software and geospatial modification to determine the users’ change in physical activity pattern, up to subjective measures, but of high quality such as the SOPARC, the IPAQ, and PARA, which are questionnaires applied by external evaluators or self-reported, some of then were for a count of number of participants and metabolic consumption Some of these are reliable and reproducible, even the general pre- and post-follow-up surveys in the population
	Reporting bias	5 studies have a protocol (Anderssen et al. 2017; Ogilvie et al. 2016; Aittasalo et al. 2019; Giles-Corti et al. 2013; Hooper et al. 2018) published before the final results, which makes it possible to establish that the bias may be present in the studies In 21, the statistical analyses used in the natural experiments to control for confounding are uni- and bivariate logistic regression, difference in differences, propensity scores, multiple regressions, and others. Also, in 5 they (Cranney et al. 2016; Jancey et al. 2016; Fitzhugh et al. 2010; Demant Klinker et al. 2015; Zhu et al. 2018) use descriptive statistical analyses and measures of mean differences between the groups

## Discussion

The results presented in this systematic review provide a better understanding of the myriad of strategies encountered in the worldwide literature regarding the promotion of PA via structural-level environmental interventions. Twenty-six studies were found that identified 4 fundamental areas of structural modifications to promote PA including schools, work environments, streets or cities, and neighborhoods or parks. Of the 26 studies, 21 examined the effect of environmental modifications like parks, cities, pedestrian walkways, or steps, while 5 examined modifications in indoor or closed environments like in schools and the workplace. A summary of the findings is presented in Supplementary material 2.

Environmental modification strategies at both the physical and social level appear to bring about changes in a subjects’ behavior, which was observed in this study. One key message from this systematic review is that structural-level environmental interventions and modifications such as providing physical space or programs to facilitate PA does in fact increase PA leading to healthier lifestyles that may decrease the development of chronic diseases (Sisson et al. 2016; Sarmiento et al. 2020). There is also evidence of a worldwide increase in PA levels and a positive association between the amount of walking and use of the bicycle in adults and older adults with the development of paths and bicycle paths, respectively, as reported in the manuscripts included in this review (Gomez et al. 2015). In the same sense, neighborhoods that facilitate spaces for walking and green spaces demonstrate a positive association with an increase in PA (Sarmiento et al. 2020). Similarly, structural modifications to promote additional standing and walking in schools and universities showed increased PA in children and young adults (Sisson et al. 2019). In addition, having outdoor spaces and infrastructures in parks, along with paths for bicycles or walking increases the use of bicycles and the development of exercise programs (Mölenberg et al. 2019b; McCormack and Shiell 2011), producing favorable changes in the level of activity, METS or time spent in PA.

These environmental modifications have been studied from different perspectives, such as the implementation of paths and different methods of transportation, changes in schools to control obesity and strategies in an open environment to promote healthy behaviors (Hirsch et al. 2018; Sarmiento et al. 2020; Sisson et al. 2016). A systematic review of the natural experiment found evidence for the modification of transportation paths to increase PA (Hirsch et al. 2018) in which changing the type of transportation resulted in an important an environmental strategy to promote PA with findings similar to our systematic review. In contrast to our study, the authors performed a meta-analysis of the available evidence and found high levels of statistical heterogeneity with I^2^ values of 98%, which is to be expected due to the differences in the protocols and the populations measured in a natural experiment.

Another study was an integrative review (Gomez et al. 2015) examining the effects of urban modifications on active transportation to promote PA. Fourteen reviews were included in the study and all related to environmental modification of spaces for transportation or bicycles to increase active rather than passive transportation. Unlike the present systematic review which focuses on natural experiments, the authors combined and analyzed a variety of study designs such as cohort, cross-sectional, experimental, case, and controlled studies. Although the current systematic review examined only natural experiments making direct comparisons between the two studies challenging, results broadly similar to ours were reached including an increase in the use of transportation and the number of travels like as active transports.

Another study of note was a systematic review of the interventions used to promote bicycle use through structural modification (Mölenberg et al. 2019b). Similar to the current systematic review, data from natural experiments were reviewed yielding a total of 31 studies. Of these 31 studies, 20 focused on structural modification in the environment and 16 examined the combined effect of the structural modification and use of the infrastructure of bicycle paths. The authors found improvements in performance outcomes measured for both the use of bicycles and bicycle paths in 11 countries studied. Of these 11 countries, 7 have similar outcomes in PA to those described in our study, while the remainder focus on the measurement of the use of bicycles rather than the effect on PA.

An additional review article related to environmental modification (McCormack and Shiell 2011), but not focused on transportation, evaluated 33 studies all of which had different methodologies that included quasi-experimental and cross-sectional designs. All of the studies examined the effect of environmental modifications on PA, but as in the present review, from the perspective of modifications to neighborhoods, land use for activity, parks, tracks and walking trails or transportation structures. All studies were found to improve walking time and active movement either by walking or cycling more and promoting an active lifestyle among the populations studied. Similar to the current systematic review, instrumental and non-instrumental measures of PA were reported in 31 of the 33 studies and 2 were excluded because such measurements were not performed. Instrumental measurements such as accelerometers and pedometers were used in 4 studies, while questionnaires on PA levels, including walking, cycling or moderate-vigorous level of activity were used in the remaining 27 studies. Although an increase in PA resulting from the environmental modification was found, an increase in the walking indices following neighborhood modifications was not consistently observed.

It is important to note that there is evidence regarding the potential influence of diet and nutrition influencing environmental modifications such as the use of supermarkets, roads and neighborhoods. A systematic review (Macmillan et al. 2018) examined the influence of diet and nutrition on PA in 15 natural experiments of which 4 were on supermarkets and foods specifically and the remaining 11 studies examined the effect of diet and nutrition on walking, footpaths, parks, green areas, and the modification of roads and byways. Like in the present systematic review, most of the studies were conducted in the United States (10 of the 15 studies), and the remainder in the United Kingdom, New Zealand and Brazil. Two of the studies used accelerometers, 3 accelerometers combined with GPS, and the remainder used questionnaires and self-reports as outcome measures. It is worth noting that a control group was included in some of the studies while the remaining studies examined outcomes pre- and post-urban remodeling. The overall results of the above study found that diet and nutrition or modifications in the environment were not effective at increasing PA.

Limitations to this study include the quality of the data due to the challenge of follow-up in certain populations as well as attrition of participants and fluctuating populations that participate voluntarily in the experiment rather than participant recruitment into a study. The examination of PA in studies that are natural experiments with their implicit risk of bias due to the lack of a control group, variability in populations and environments, different structural interventions, follow-up and attrition of subjects, and population migration. Regarding the risk of bias we chose to assess the risk of bias and its impact on the results of the studies examining the effect of environmental modifications on PA using the criteria of the ROBINS I instrument for non-randomized studies (Benton et al. 2016). We assessed the risk of bias, based on the previously outlined approach, and found a moderate to severe risk in many of the criteria due to the nature of natural experimentation. However, we decided to take the criteria from the list for each of the phases but not the qualifiers, which differentiates the risk analysis in this study compared to others. Therefore, placing the qualitative emphasis, according to the design, on its importance for public health and decision-making in health policy and care.

One significant strength of the study is the measurement of several specific interventions in large groups of individuals in different parts of the world. In addition, unusual community-level interventions have been employed and examined in the included studies such as the creation of cycle lanes, bicycle paths, outdoor gyms, and others.

Another important aspect is that the current study found that the instrumental methods used and analysis of outcomes described in the reviewed studies are consistent with management guidelines for natural experiments according to the Research Council (Craig et al. 2012). The suggested analyses for such designs including logistic regression, the difference in differences, propensity scores and multiple regressions, are present in 80% of the studies that comprise this review. Thus, we provide a more robust and comprehensive systematic review examining the effect of structural-level environmental interventions on PA.

## Conclusion

The results of the above systematic review highlight the important role that natural experiments have on decision-making in public health and health care, especially in regards to the quality of life and reduction of risk factors by means of PA through environmental interventions. Based on the results of this systematic review it seems prudent that a natural experimental study considers controlling for confounding variables and establishing outcome measures that can readily identify the impact of interventions on health indicators in the countries where they are conducted to incorporate actions and public policy guidelines in such countries and worldwide.

As with any study based on the results from natural experiments, there are potential limitations in the evaluation of data. However, at the same time, we believe that from a public health perspective this type of study plays an important role in ensuring the continued application of such experiments in real-life situations. The current systematic review suggests that modification of the environment is an important element in improving public health through increased PA for which reason we encourage the use of natural experiments to examine such interventions.

Based on our systematic review the environmental interventions of structural modification in parks and active transportation demonstrated greater effectiveness in increasing the level and time of PA in the population study.

In schools and work areas, there is evidence of both a decrease in sedentary time and an increase in PA related to environmental modification of space or change in office structure.

Finally, given that the economic and cultural setting is a key variable when considering the effectiveness of structural interventions, and since only 1 of the 26 reviewed articles included such data, more studies examining economic factors are needed especially in low- and middle-income countries like in South America.

## Supplementary Information

Below is the link to the electronic supplementary material.

Supplementary file1 (DOCX 16 KB)

Supplementary file2 (DOCX 35 KB)

## Data Availability

The data included in this review are in the possession of the researchers and are freely accessible to those who submit a justified request.

## Abbreviations

PA: Physical activity
COPD: Chronic obstructive pulmonary disease
PRISMA: Preferred Reporting Items for Systematic Reviews and Meta-Analyses
PROSPERO: International Database of Prospectively Registered Systematic Reviews
IPAQ: International Physical Activity Questionnaire
CHAMPS: System outlines expected behavior for students in each activity throughout the daily
Kcal/Kg: Kilocalorie/kilograms
Kcal: Kilocalorie
METS: Metabolic equivalents
CO_2_/*V*O_2_: Carbon dioxide/oxygen consumption
CINAHL: Cumulative Index of Nursing and Allied Literature
Mesh: Medical subject headings
Dec’s: Health descriptors
Entrée: Embase subject headings
ROBINS I: Risk of bias tool to assess non-randomized studies of interventions I
GPS: Global positioning system
ACTIVE PAL 3C: Active physical activity location measurement system
PALMS: Physical activity location measurement system
SOPARC: System to assess the practice of physical activity and recreation in parks and natural surroundings
IPAQ: International Physical Activity Questionnaire
RPARQ: Recreation Physical Activity Questionnaire
OR: Odds ratio
CI: Confidence interval
RRR: Rate risk ratio
I2: Statistic for heterogeneity
UAB: Universitat Autònoma de Barcelona
LPA: Light physical activity
MPA: Moderate physical activity
MVPA: Moderate-vigorous physical activity
VPA: Vigorous physical activity
