# Supplementary material for: Impact of structural-level environmental interventions on physical activity: a systematic review
Source: Int Arch Occup Environ Health. 2023 Apr 26;96(6):815–38. doi: 10.1007/s00420-023-01973-w (PMC10272243; doi:10.1007/s00420-023-01973-w)
Supplement: Supplementary file 1 — Supplementary file1 (DOCX 16 KB) [file 420_2023_1973_MOESM1_ESM.docx]

search strategy

PUBMED

#1 "Physical Fitness"[Mesh]

#2 "Exercise"[Mesh]

#3 "Physical Exertion"[Mesh]

# 4 "Sports"[Mesh]

# 5 "Walking"[Mesh]

# 6 “physical activity”

# 7 Bicycling

# 8 "activity habits"

# 9 cycleway

# 10 park

# 11 healthy park

# 12 active transport to school

# 13 built environment

# 14 Active travel

# 15 Infrastructure

(((((((((((((Physical Fitness) OR physical activity) OR physical exercise) OR exercises) OR Physical Exertion) OR Walking) OR Bicycling) OR "activity habits") OR cycleway) OR park) OR active transport to school) OR built environment) OR Active travel) OR Infrastructure

(((((((((((("physical fitness"[All Fields]) OR (Exercise)) OR (Physical Exertion)) OR (Sports)) OR (Walking)) OR (physical activity)) OR (Bicycling)) OR (activity habits)) OR (cycleway)) OR ("park"[All Fields])) OR (healthy park)) OR (active transport school)) OR (((((((citizen science) ) OR (community engagement)) OR (architecture)) OR (built environment)) OR (Active travel)) OR (Infrastructure))

((((((((("Health Promotion"[Mesh]) OR "Health Education"[Mesh]) OR "Patient Education as Topic"[Mesh]) OR "Primary Prevention"[Mesh]) OR "Primary Health Care"[Mesh]) OR "Public Policy"[Mesh]) OR "Program") OR “education program”) OR “community education”) OR “life styles”

 ((((((((((((("physical fitness"[All Fields]) OR (Exercise)) OR (Physical Exertion)) OR (Sports)) OR (Walking)) OR (physical activity)) OR (Bicycling)) OR (activity habits)) OR (cycleway)) OR ("park"[All Fields])) OR (healthy park)) OR (active transport school)) OR (((((((citizen science) ) OR (community engagement)) OR (architecture)) OR (built environment)) OR (Active travel)) OR (Infrastructure))) AND (((((((((("Health Promotion"[Mesh]) OR "Health Education"[Mesh]) OR "Patient Education as Topic"[Mesh]) OR "Primary Prevention"[Mesh]) OR "Primary Health Care"[Mesh]) OR "Public Policy"[Mesh]) OR "Program") OR "education program") OR "community education") OR "life styles")

("physical fitness"[All Fields] OR ("exercise"[MeSH Terms] OR "exercise"[All Fields] OR "exercises"[All Fields] OR "exercise therapy"[MeSH Terms] OR ("exercise"[All Fields] AND "therapy"[All Fields]) OR "exercise therapy"[All Fields] OR "exercise s"[All Fields] OR "exercised"[All Fields] OR "exerciser"[All Fields] OR "exercisers"[All Fields] OR "exercising"[All Fields]) OR ("physical exertion"[MeSH Terms] OR ("physical"[All Fields] AND "exertion"[All Fields]) OR "physical exertion"[All Fields]) OR ("sport s"[All Fields] OR "sports"[MeSH Terms] OR "sports"[All Fields] OR "sport"[All Fields] OR "sporting"[All Fields]) OR ("walked"[All Fields] OR "walking"[MeSH Terms] OR "walking"[All Fields] OR "walks"[All Fields]) OR ("exercise"[MeSH Terms] OR "exercise"[All Fields] OR ("physical"[All Fields] AND "activity"[All Fields]) OR "physical activity"[All Fields]) OR ("bicycle"[All Fields] OR "bicycled"[All Fields] OR "bicycles"[All Fields] OR "bicycling"[MeSH Terms] OR "bicycling"[All Fields]) OR (("activable"[All Fields] OR "activate"[All Fields] OR "activated"[All Fields] OR "activates"[All Fields] OR "activating"[All Fields] OR "activation"[All Fields] OR "activations"[All Fields] OR "activator"[All Fields] OR "activator s"[All Fields] OR "activators"[All Fields] OR "active"[All Fields] OR "actived"[All Fields] OR "actively"[All Fields] OR "actives"[All Fields] OR "activities"[All Fields] OR "activity s"[All Fields] OR "activitys"[All Fields] OR "motor activity"[MeSH Terms] OR ("motor"[All Fields] AND "activity"[All Fields]) OR "motor activity"[All Fields] OR "activity"[All Fields]) AND ("habits"[MeSH Terms] OR "habits"[All Fields] OR "habit"[All Fields])) OR "cycleway"[All Fields] OR "park"[All Fields] OR (("healthies"[All Fields] OR "healthy"[All Fields]) AND "park"[All Fields]) OR (("biological transport, active"[MeSH Terms] OR ("biological"[All Fields] AND "transport"[All Fields] AND "active"[All Fields]) OR "active biological transport"[All Fields] OR ("active"[All Fields] AND "transport"[All Fields]) OR "active transport"[All Fields]) AND ("educational status"[MeSH Terms] OR ("educational"[All Fields] AND "status"[All Fields]) OR "educational status"[All Fields] OR "schooling"[All Fields] OR "education"[MeSH Terms] OR "education"[All Fields] OR "school s"[All Fields] OR "schooled"[All Fields] OR "schools"[MeSH Terms] OR "schools"[All Fields] OR "school"[All Fields])) OR ("citizen science"[MeSH Terms] OR ("citizen"[All Fields] AND "science"[All Fields]) OR "citizen science"[All Fields] OR (("communal"[All Fields] OR "communalism"[All Fields] OR "communalities"[All Fields] OR "communality"[All Fields] OR "communally"[All Fields] OR "commune"[All Fields] OR "communes"[All Fields] OR "community s"[All Fields] OR "communitys"[All Fields] OR "residence characteristics"[MeSH Terms] OR ("residence"[All Fields] AND "characteristics"[All Fields]) OR "residence characteristics"[All Fields] OR "communities"[All Fields] OR "community"[All Fields]) AND ("engage"[All Fields] OR "engaged"[All Fields] OR "engagement s"[All Fields] OR "engagements"[All Fields] OR "engages"[All Fields] OR "engaging"[All Fields] OR "social participation"[MeSH Terms] OR ("social"[All Fields] AND "participation"[All Fields]) OR "social participation"[All Fields] OR "engagement"[All Fields])) OR ("architectural"[All Fields] OR "architecturally"[All Fields] OR "architecture"[MeSH Terms] OR "architecture"[All Fields] OR "architecture s"[All Fields] OR "architectured"[All Fields] OR "architectures"[All Fields]) OR ("built environment"[MeSH Terms] OR ("built"[All Fields] AND "environment"[All Fields]) OR "built environment"[All Fields]) OR (("activable"[All Fields] OR "activate"[All Fields] OR "activated"[All Fields] OR "activates"[All Fields] OR "activating"[All Fields] OR "activation"[All Fields] OR "activations"[All Fields] OR "activator"[All Fields] OR "activator s"[All Fields] OR "activators"[All Fields] OR "active"[All Fields] OR "actived"[All Fields] OR "actively"[All Fields] OR "actives"[All Fields] OR "activities"[All Fields] OR "activity s"[All Fields] OR "activitys"[All Fields] OR "motor activity"[MeSH Terms] OR ("motor"[All Fields] AND "activity"[All Fields]) OR "motor activity"[All Fields] OR "activity"[All Fields]) AND ("travel"[MeSH Terms] OR "travel"[All Fields] OR "traveling"[All Fields] OR "travelling"[All Fields] OR "travels"[All Fields] OR "traveled"[All Fields] OR "traveler"[All Fields] OR "traveler s"[All Fields] OR "travelers"[All Fields] OR "travelled"[All Fields] OR "traveller"[All Fields] OR "traveller s"[All Fields] OR "travellers"[All Fields])) OR ("infrastructural"[All Fields] OR "infrastructure"[All Fields] OR "infrastructures"[All Fields]))) AND ("Health Promotion"[MeSH Terms] OR "Health Education"[MeSH Terms] OR "Patient Education as Topic"[MeSH Terms] OR "Primary Prevention"[MeSH Terms] OR "Primary Health Care"[MeSH Terms] OR "Public Policy"[MeSH Terms] OR "Program"[All Fields] OR "education program"[All Fields] OR "community education"[All Fields] OR "life styles"[All Fields])

Translations

Exercise: "exercise"[MeSH Terms] OR "exercise"[All Fields] OR "exercises"[All Fields] OR "exercise therapy"[MeSH Terms] OR ("exercise"[All Fields] AND "therapy"[All Fields]) OR "exercise therapy"[All Fields] OR "exercise's"[All Fields] OR "exercised"[All Fields] OR "exerciser"[All Fields] OR "exercisers"[All Fields] OR "exercising"[All Fields]

Physical Exertion: "physical exertion"[MeSH Terms] OR ("physical"[All Fields] AND "exertion"[All Fields]) OR "physical exertion"[All Fields]

Sports: "sport's"[All Fields] OR "sports"[MeSH Terms] OR "sports"[All Fields] OR "sport"[All Fields] OR "sporting"[All Fields]

Walking: "walked"[All Fields] OR "walking"[MeSH Terms] OR "walking"[All Fields] OR "walks"[All Fields]

physical activity: "exercise"[MeSH Terms] OR "exercise"[All Fields] OR ("physical"[All Fields] AND "activity"[All Fields]) OR "physical activity"[All Fields]

Bicycling: "bicycle"[All Fields] OR "bicycled"[All Fields] OR "bicycles"[All Fields] OR "bicycling"[MeSH Terms] OR "bicycling"[All Fields]

activity: "activable"[All Fields] OR "activate"[All Fields] OR "activated"[All Fields] OR "activates"[All Fields] OR "activating"[All Fields] OR "activation"[All Fields] OR "activations"[All Fields] OR "activator"[All Fields] OR "activator's"[All Fields] OR "activators"[All Fields] OR "active"[All Fields] OR "actived"[All Fields] OR "actively"[All Fields] OR "actives"[All Fields] OR "activities"[All Fields] OR "activity's"[All Fields] OR "activitys"[All Fields] OR "motor activity"[MeSH Terms] OR ("motor"[All Fields] AND "activity"[All Fields]) OR "motor activity"[All Fields] OR "activity"[All Fields]

habits: "habits"[MeSH Terms] OR "habits"[All Fields] OR "habit"[All Fields]

healthy: "healthies"[All Fields] OR "healthy"[All Fields]

active transport: "biological transport, active"[MeSH Terms] OR ("biological"[All Fields] AND "transport"[All Fields] AND "active"[All Fields]) OR "active biological transport"[All Fields] OR ("active"[All Fields] AND "transport"[All Fields]) OR "active transport"[All Fields]

school: "educational status"[MeSH Terms] OR ("educational"[All Fields] AND "status"[All Fields]) OR "educational status"[All Fields] OR "schooling"[All Fields] OR "education"[MeSH Terms] OR "education"[All Fields] OR "school's"[All Fields] OR "schooled"[All Fields] OR "schools"[MeSH Terms] OR "schools"[All Fields] OR "school"[All Fields]

citizen science: "citizen science"[MeSH Terms] OR ("citizen"[All Fields] AND "science"[All Fields]) OR "citizen science"[All Fields]

community: "communal"[All Fields] OR "communalism"[All Fields] OR "communalities"[All Fields] OR "communality"[All Fields] OR "communally"[All Fields] OR "commune"[All Fields] OR "communes"[All Fields] OR "community's"[All Fields] OR "communitys"[All Fields] OR "residence characteristics"[MeSH Terms] OR ("residence"[All Fields] AND "characteristics"[All Fields]) OR "residence characteristics"[All Fields] OR "communities"[All Fields] OR "community"[All Fields]

engagement: "engage"[All Fields] OR "engaged"[All Fields] OR "engagement's"[All Fields] OR "engagements"[All Fields] OR "engages"[All Fields] OR "engaging"[All Fields] OR "social participation"[MeSH Terms] OR ("social"[All Fields] AND "participation"[All Fields]) OR "social participation"[All Fields] OR "engagement"[All Fields]

architecture: "architectural"[All Fields] OR "architecturally"[All Fields] OR "architecture"[MeSH Terms] OR "architecture"[All Fields] OR "architecture's"[All Fields] OR "architectured"[All Fields] OR "architectures"[All Fields]

built environment: "built environment"[MeSH Terms] OR ("built"[All Fields] AND "environment"[All Fields]) OR "built environment"[All Fields]

Active: "activable"[All Fields] OR "activate"[All Fields] OR "activated"[All Fields] OR "activates"[All Fields] OR "activating"[All Fields] OR "activation"[All Fields] OR "activations"[All Fields] OR "activator"[All Fields] OR "activator's"[All Fields] OR "activators"[All Fields] OR "active"[All Fields] OR "actived"[All Fields] OR "actively"[All Fields] OR "actives"[All Fields] OR "activities"[All Fields] OR "activity's"[All Fields] OR "activitys"[All Fields] OR "motor activity"[MeSH Terms] OR ("motor"[All Fields] AND "activity"[All Fields]) OR "motor activity"[All Fields] OR "activity"[All Fields]

travel: "travel"[MeSH Terms] OR "travel"[All Fields] OR "traveling"[All Fields] OR "travelling"[All Fields] OR "travels"[All Fields] OR "traveled"[All Fields] OR "traveler"[All Fields] OR "traveler's"[All Fields] OR "travelers"[All Fields] OR "travelled"[All Fields] OR "traveller"[All Fields] OR "traveller's"[All Fields] OR "travellers"[All Fields]

Infrastructure: "infrastructural"[All Fields] OR "infrastructure"[All Fields] OR "infrastructures"[All Fields]
