# Supplementary material for: Impact of structural-level environmental interventions on physical activity: a systematic review
Source: Int Arch Occup Environ Health. 2023 Apr 26;96(6):815–38. doi: 10.1007/s00420-023-01973-w (PMC10272243; doi:10.1007/s00420-023-01973-w)
Supplement: Supplementary file 2 — Supplementary file2 (DOCX 35 KB) [file 420_2023_1973_MOESM2_ESM.docx]

Appendix 2 Narrative summary of findings about the effectiveness of the structural modifications by outcome measure.

| Area or outcome | Finding |
| --- | --- |
| **activity level by percentage or minutes** | - Increase in activity time between 10 and 30 minutes of total - Increase 6 to 28% or between 38 and 369 users - Parks area with outdoor gyms more effective and significant - Light physical activity increased from 22 and 67 minutes or 6 and 8%. - Vigorous level, no increase is reported in 80% of the studies and the remaining 20% - Major difference in work and transportation, greater times of light and moderate activity. - 54% increase recreational walking with an increase in minutes/week post-renovation. - Increase in moderate-vigorous activity in the area of the new neighborhood. |
| **Counts** | - Increase in participation counts in parks and active transport in bicycle lanes. - Reduction in the counts in system of boulevards for active transport. - Increase the number of participants in modification of parks with outdoor gyms and areas for recreation and exercise - Increase 45% for the activities of walking, cycling and running in the park post-remodeling construction of a green area in the city OR 1.46 CI 1.06 – 1.98 . |
| **Metabolic Consumption and Steps Number** | - Increase in metabolic work METS in parks, schools and area of transportation. - The time spent sitting versus active time in the environmental programs decrease sitting and increase level of activity - Work studies reduction in sitting time or of seated work, increase in activity standing up or an increase in light physical activity. - Slight increase in time in minutes of 130 to 150 minutes in light activity - No increase in moderate-vigorous activity in the work areas, - School, reduction in sitting time in minutes of the students’ day. - Increase number of steps with pedometers or accelerometers. |
| **Cities** | - Increase in activity in the population reached the light activity goals OR 1.46 CI from 1.11 to 1.92 in men with a higher education level. - Program lasts longer than 6 months, it increases the OR - increase activity in children and young people after urban renovation and modification of spaces in cities. - Increase in activity post-renovation with greater activity in minutes/week in the green areas and transportation after a school activity - Greater light activity is evidenced globally in minutes of activity in the population attending the activity. - After urban modification increase 7.8 minutes in light activity, 4.5 minutes moderate-vigorous, and 13.1 minutes less in sitting time with an increase in play time for students over 40 minutes/week. |
| **Commuting** | - Increase in the number of users and the activity, especially if the users live near the travel area - Increase in physical activity from school to home by bicycle or walking from school in the neighborhood - increase in the number of trips and users going by bicycle at occupational level - increase in active transport for taking the subway with a reduction in bus time and an increase in bicycle time - changed from car to bicycle with an increase in minutes by bicycle use and a reduction in car use with in commute time from 10 to 150 minutes per week with a rate risk ratio RRR 2.5 CI 1.2 to 5.0. - Greater metabolic consumption measured in METS for the use of the bicycle and walking in the moderate-vigorous activity and increase 150 minutes/week of walking. |
